# Alterations of the White Matter in Patients With Knee Osteoarthritis: A Diffusion Tensor Imaging Study With Tract-Based Spatial Statistics

**DOI:** 10.3389/fneur.2022.835050

**Published:** 2022-03-17

**Authors:** Shirui Cheng, Xiaohui Dong, Jun Zhou, Chenjian Tang, Wenhua He, Yang Chen, Xinyue Zhang, Peihong Ma, Tao Yin, Yimei Hu, Fang Zeng, Zhengjie Li, Fanrong Liang

**Affiliations:** ^1^The Acupuncture and Tuina School, Chengdu University of Traditional Chinese Medicine, Chengdu, China; ^2^The First Affiliated Hospital, Chengdu University of Traditional Chinese Medicine, Chengdu, China; ^3^The Second Affiliated Hospital of Shanxi, University of Traditional Chinese Medicine, Taiyuan, China; ^4^Acupuncture and Moxibustion Department, Beijing University of Chinese Medicine, Beijing, China; ^5^Clinical Medical School, Chengdu University of Traditional Chinese Medicine, Chengdu, China

**Keywords:** knee osteoarthritis (KOA), diffusion tensor imaging, white matter, tract-based spatial statistics, neuroimaging

## Abstract

**Background:**

Functional and structural alterations in the gray matter have been observed in patients with knee osteoarthritis (KOA). However, little is known about white matter changes in KOA. Here, we evaluated fractional anisotropy (FA), mean diffusivity (MD), axial diffusivity (AD), and radial diffusivity (RD) to investigate potential alterations in the white matter of patients with KOA.

**Methods:**

A total of 166 patients with KOA, along with 88 age- and sex-matched healthy controls were recruited and underwent brain magnetic resonance imaging (MRI). Diffusion tensor imaging (DTI) data were collected and analyzed using tract-based spatial statistics (TBSS). Statistical significances were determined at *p* < 0.05 and were corrected by the threshold-free cluster enhancement (TFCE) method. Then, we evaluated potential correlations between FA, MD, AD, RD values and disease duration, Western Ontario and McMaster Universities Osteoarthritis Index (WOMAC) scores, and visual analog scale (VAS) scores.

**Results:**

FA values for the body of corpus callosum, splenium of corpus callosum, bilateral superior longitudinal fasciculus, cingulum, bilateral superior corona radiata, and right posterior corona radiata were significantly higher in patients with KOA than in healthy controls (*p* < 0.05, TFCE corrected). Compared with healthy controls, patients with KOA also had significantly lower MD, AD, and RD values of the genu of corpus callosum, body of corpus callosum, splenium of corpus callosum, corona radiata, right posterior thalamic radiation, superior longitudinal fasciculus, and middle cerebellar peduncle (*p* < 0.05, TFCE corrected). Negative correlations were detected between WOMAC scores and AD values for the body of the corpus callosum and the splenium of the corpus callosum (*p* < 0.05, FDR corrected).

**Conclusion:**

Patients with KOA exhibited extensive white matter alterations in sensorimotor and pain-related regions. Longitudinal observation studies on the causation between abnormalities in the white matter tracts and KOA is needed in the future.

## Introduction

Osteoarthritis (OA) is a common cause of pain and disability in the elderly (1) and predominantly affects the knee joint (2). Knee osteoarthritis (KOA) affects at least 15–18% of people globally (3), reduces multiple facets of the quality of life (QOL), and induces an enormous healthcare burden in industrialized societies (4). The risk factors for developing knee osteoarthritis are age, obesity, and articular malalignment (2). According to clinical guidelines, the first therapeutic principle for is to relieve knee pain (5). However, the pathology of KOA is not well-understood, thus restricting the development of specific therapeutic protocols for clinical practice.

It is generally believed that the key factor underlying KOA is inflammation due to the breakdown of joint tissues from mechanical loading, aging, or other factors (6, 7). However, these peripheral abnormalities do not fully account for the intensity of pain in patients with chronic musculoskeletal pain (8) because substantial discordance exists between radiographic OA of the knee when compared to knee pain (9, 10). With the development of neuroimaging techniques, researchers have found that the central neural system plays a key role in KOA (11). For example, several recent studies reported abnormal functions of the gray matter in the lateral prefrontal cortex, parietal lobule, anterior cingulate cortex, insula and limbic cortical, which were involved in altered pain processing in KOA patients (12–14). These findings were further validated by the observation of structural changes in the gray matter in other neuroimaging studies (15, 16). Given that the observed alterations in the structure and function of the gray matter arise from the adaption or maladaption of the brain to certain conditions such as prolonged nociceptive input from chronic knee pain, it is reasonable to hypothesize that the white matter could also be affected by this condition. However, little is known about white matter alterations in patients with KOA.

Diffusion tensor imaging (DTI) can provide significant insight into the diffusion of water molecules and thus quantify microstructural alterations within the white matter (17, 18). Tract-based spatial statistics (TBSS) is the most common method used to analyze DTI data (19) and includes four metrics: fractional anisotropy (FA), axial diffusivity (AD), radial diffusivity (RD), and mean diffusivity (MD). FA, as a marker of axonal membrane circumference and packing density, reflects the orientation and distribution of the random movements of water-molecules (20). AD can reflect diffusional directionality along axons and is related to the degree of myelination in the white matter (21). RD can characterize the diffusional directionality perpendicular to axons and is related to the beginning of demyelination (22) or axonal damage (20). MD reflects the diffusion magnitude; this is related to inflammation and edema in the white matter tracts (20). DTI and TBSS have been used wildly for detecting abnormal white matter in various disorders, such as schizophrenia spectrum disorders (23), chronic back pain (24), osteoarthritis (13, 14), and fibromyalgia syndrome (FMS) (25). In this study, we used the whole-brain TBSS method to investigate potential differences in the white matter tracts of patients with KOA and compared data with that derived from healthy controls (HCs). We also correlated abnormal FA, MD, AD, and RD values with clinical variables in patients with KOA to assess the clinical meaning of our findings.

## Materials and Methods

### Participants

Patients with a diagnosis of KOA at three hospitals (The First Affiliated Hospital of Chengdu University of Traditional Chinese Medicine, the Third Affiliated Hospital of Chengdu University of Traditional Chinese Medicine, and the Orthopedic Hospital of Sichuan Province) were enrolled from September 2016 to September 2021. Brain magnetic resonance imaging (MRI) scans were obtained on a GE 3.0T MRI scanner (GE 3.0T MR750, Wauwatosa, WI) using a 16-channel head coil in Chengdu, China. Age- and sex-matched HCs were also recruited. This study was carried out in accordance with the Declaration of Helsinki and was approved by the Institutional Review Board and Ethics Committees of the First Affiliated Hospital of Chengdu University of Traditional Chinese Medicine (No. 2016KL-017).

The diagnostic symptoms and signs of KOA patients were assessed by two experienced orthopedists according to the American College of Rheumatology (ACR) criteria (26). Patients with KOA were recruited if they met the following inclusion criteria: (a) aged from 38 to 70 years and right-handed; (b) were diagnosed with KOA; (c) had a pain intensity >3 on a 10-point numeric scale; (d) had a knee joint radiological degree on the Kellgren-Lawrence scale of 0-II (27); and (e) had signed a written and informed consent form. Patients with KOA were excluded if they (a) had other major painful, psychiatric, or neurological diseases; (b) had drug or alcohol addiction; (c) had contraindications for MRI scans; (d) had taken any pain killer medicine or complementary and alternative therapies within the previous month; or (e) were pregnant or lactating.

HCs were recruited if they met the following inclusion criteria: (a) aged from 38 to 70 years and right-handed; (b) were free from any pain disorders; and (c) signed the written and informed consent form. HCs were excluded if they met the following exclusion criteria: (a) accompanied by rheumatoid arthritis, high blood pressure, diabetes, or psychiatric or neurological diseases; (b) had drug or alcohol addiction; (c) had contraindications for MRI scans; (d) had taken any medicine or complementary and alternative therapies within the previous month; or (e) were pregnant or lactating.

### Clinical Data Acquisition

A range of data were collected for each patient, including age, gender, height, weight, education level, and disease duration. The average intensity of pain over the previous 2 weeks was also obtained from all KOA participants using the visual analog scale (VAS). The Western Ontario and McMaster Universities Osteoarthritis Index (WOMAC) was used to assess the symptoms and QOL of the KOA patients. Anxiety and depression were evaluated in the KOA patients by using the validated Chinese version of the self-rating anxiety scale (SAS) and self-rating depression scale (SDS). The VAS, WOMAC, SAS, and SDS assessments were administered on the same day when the MRI scan was performed.

### Image Acquisition

Brain MRI scanning sequences including three-dimensional T1-weighted (3DT1) MRI scans and diffusion-weighted DTI sequence with single-shot echo-planar imaging were performed for all participants at baseline. The parameters of the 3DT1 scans were as follows: repetition time (TR) = 6.008 ms, echo time (TE) = 1.7 ms, data matrix = 256 × 256, field of view (FOV) = 256 × 256 mm^2^, and voxel size = 1.0 × 1.0 × 1.0 mm^3^. The parameters of DTI scans were: FOV = 256 × 256 mm^2^, TR = 8,500 ms, echo time = minimum, matrix = 128 × 128, number of diffusion-encoding directions = 64, slice thickness 2 mm, layer spacing = 0, and gradient values b = 0 s/mm^2^ and b = 1,000 s/mm^2^.

### Diffusion Data Process

DTI data preprocessing and statistical analysis were conducted using the FMRIB software library (FSL; http://www.fmrib.ox.ac.uk/fsl/) (28). Data preprocessing steps included correction for eddy current effects and head motion using FDT (FMRIB's Diffusion Toolbox), extraction of the brain mask with FSL's brain extraction tool (BET), and the calculation of diffusion tensors by the DTIFIT program. After preprocessing, tract-based spatial statistical analysis was performed, including non-linear registration of each participant's FA image to a 1 × 1 × 1 mm^3^ standard space of the FMRIB58-FA template. These images were affine co-registered to the MNI152 standard space, and tracts were averaged to create a mean FA skeleton, extracting the FA skeleton, and projecting each participant's aligned FA image back onto the mean FA skeleton with a 0.2 FA threshold. The MD, AD, and RD images of individual participants were also projected onto the mean FA skeleton.

### Statistical Analysis

Statistical comparison of the clinical data between patients with KOA and HCs was performed using SPSS Statistics version 22.0 (IBM Corp., Armonk, NY). Age and body mass index (BMI) were compared between the two groups using a non-parametric test. Gender distribution was analyzed between the two groups using the *chi*-squared test.

Voxel-brain skeletal FA, MD, AD, and RD analysis was performed between the KOA patients and HCs using a general linear model through the FSL randomize toolkit. Age, gender, and BMI were used as covariates. A 5,000-repetition permutation test was conducted between the KOA patients and HCs, and significant clusters were corrected by the threshold-free cluster enhancement method (TFCE, *p* < 0.05). After correction, only clusters with voxel size >100 were reported (29). JHU ICBM-DTI-81 White-Matter Labels in FSL were used to identify white matter tracts showing significant alterations. Spearman's correlation analysis was conducted between the FA, MD, AD, and RD values of significant clusters and a range of clinical characteristics including disease duration, VAS scores, and WOMAC scores, which were corrected by the false discovery rates method (FDR, *p* < 0.05).

## Results

### Clinical Characteristics

A total of 166 patients with a diagnosis of KOA (125 females, age range: 39–67 years, mean ± SD: 52.87 ± 5.23 years) and 88 HCs (56 females, age range: 42–62 years, mean ± SD: 53.76 ± 4.82 years) were recruited in this study. There were no significant differences between the two groups in terms of age, gender, and BMI (*p* > 0.05). The mean duration of patients with KOA was 45.95 ± 50.18 months (range: 1–241 months) and the mean WOMAC and VAS scores of patients with KOA were 35.73 ± 28.63 and 4.31 ± 1.31, respectively. The demographic and clinical data of the KOA patients and HCs are summarized in Table 1.

**Table 1:** Clinical and demographic characteristics of KOA and HC.

**Items**	**KOA**	**HS**	***P*-value**
	**(*n* = 166)**	**(*n* = 88)**	
Age (years)	52.87 ± 5.23	53.76 ± 4.82	0.110
Gender (female/male)	125/41	56/32	0.051
BMI (kg/m^2^)	23.95 ± 2.85	23.85 ± 2.80	0.843
Duration (months)	45.95 ± 50.18	–	–
WOMAC	35.73 ± 28.63	–	–
VAS	4.31 ± 1.31	–	–
SAS	35.96 ± 8.03	–	–
SDS	30.97 ± 5.83	–	–

*Data were expressed as the Mean ± SD. BMI, body mass index; HC, health control; KOA, knee osteoarthritis; SAS, self-rating anxiety scale; SDS, self-rating depression scale; VAS, visual analog scale; WOMAC, Western Ontario and McMaster Universities Osteoarthritis Index*.

### Tract-Based Spatial Statistics Analysis

**Figure 1:**
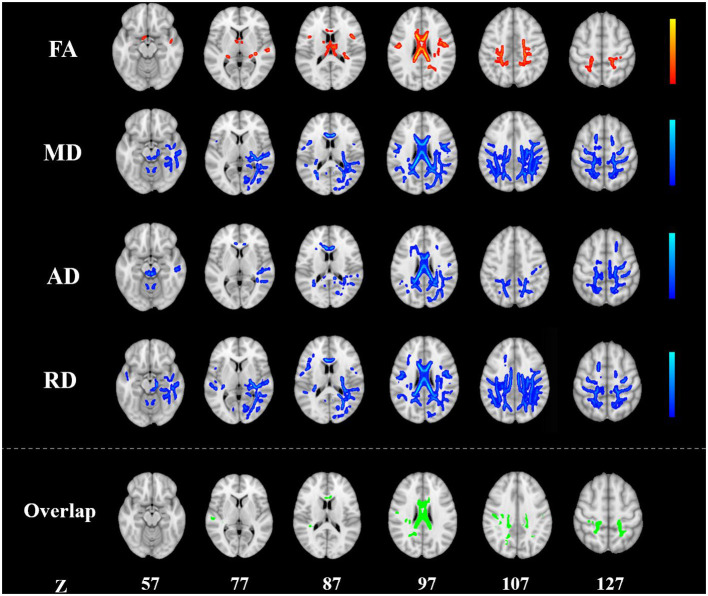
Alterations in TBSS parameters in patients with KOA. White matter regions showing increased FA and decreased MD, AD, and RD values in patients with KOA compared to healthy controls. White matter regions showing overlapping FA, MD, AD, and RD values in the body, the splenium of the corpus callosum (*p* < 0.05, TFCE corrected). AD, radial diffusivity; FA, fractional anisotropy; KOA, knee osteoarthritis; MD, mean diffusivity; RD, radial diffusivity; TBSS, tract-based spatial statistics; TFCE, threshold-free cluster enhancement.

Compared with HCs, patients with KOA showed a significant increased FA in the body of the corpus callosum (CC), splenium of CC, bilateral superior corona radiata, right posterior corona radiata, bilateral superior longitudinal fasciculus (SLF), left cingulum (cingulate gyrus), and bilateral fornix/stria terminalis (*p* < 0.05, TFCE corrected; Figure 1; Table 2).

**Table 2:** Regions with significantly increased FA and decreased MD, AD, and RD in patients with KOA.

**WM** **(JHU WM)**		**Cluster size**				
		**FA**	**MD**	**AD**	**RD**	**Overlap**
Middle cerebellar peduncle		–	905	1,068	1,179	–
Pontine crossing tract (a part of MCP)		–	–	202	–	–
Genu of corpus callosum		–	396	511	258	–
Body of corpus callosum		1,469	2,764	1,882	2,532	953
Splenium of corpus callosum		156	1,113	995	867	119
Fornix (column and body of fornix)		152	–	–	–	–
Corticospinal tract	R	–	–	131	–	–
Medial lemniscus	R	–	–	177	–	–
Medial lemniscus	L	–	–	108	–	–
Inferior cerebellar peduncle	R	–	–	154	–	–
Inferior cerebellar peduncle	L	–	–	161	–	–
Superior cerebellar peduncle	R	–	–	115	–	–
Superior cerebellar peduncle	L	–	–	131	–	–
Cerebral peduncle	R	–	203	–	283	–
Posterior limb of internal capsule	R	–	349	–	361	–
Retrolenticular part of internal capsule	R	–	641	–	661	–
Retrolenticular part of internal capsule	L	–	–	–	189	–
Anterior corona radiata	L	–	–	152	–	–
Superior corona radiata	R	146	566	143	601	
Superior corona radiata	L	139	141	–	318	–
Posterior corona radiata	R	151	736	493	524	
Posterior corona radiata	L	–	443	161	378	–
Posterior thalamic radiation (include optic radiation)	R	–	745	152	773	–
Sagittal stratum (include inferior longitudinal fasciculus and inferior fronto-occipital fasciculus)	R	–	264	–	232	–
External capsule	R	–	113	–	137	–
Cingulum (cingulate gyrus)	R	–	–	–	180	–
Cingulum (cingulate gyrus)	L	233	104	–	386	–
Cingulum (hippocampus)	R	–	113	–	180	–
Fornix (cres) / Stria terminalis	R	223	274	–	249	–
Fornix (cres) / Stria terminalis	L	143	–	–	–	–
Superior longitudinal fasciculus	R	516	1,170	296	1,073	–
Superior longitudinal fasciculus	L	100	292	–	558	–

*p < 0.05, TFCE corrected. AD, radial diffusivity; FA, fractional anisotropy; JHU WM, JHU ICBM-DTI-81 White-Matter Labels; KOA, knee osteoarthritis; L, left; MCP, middle cerebellar peduncle; MD, mean diffusivity; R, right; RD, radial diffusivity; WM, white matter*.

The MD was significantly reduced in the middle cerebellar peduncle, genu of CC, body of CC, splenium of CC, right cerebral peduncle, right posterior limb of internal capsule, right retrolenticular part of the internal capsule, bilateral superior corona radiata, bilateral posterior corona radiata, right posterior thalamic radiation, right sagittal stratum, right external capsule, left cingulum (cingulate gyrus), right cingulum (hippocampus), and bilateral SLF in KOA patients (*p* < 0.05, TFCE corrected; Figure 1; Table 2).

KOA patients had a reduced AD in the middle cerebellar peduncle, pontine corticospinal tract, genu of CC, body of CC, splenium of CC, right corticospinal tract, bilateral medial lemniscus, bilateral inferior cerebellar peduncle, bilateral superior cerebellar peduncle, left anterior corona radiata, right superior corona radiata, bilateral posterior corona radiata, right posterior thalamic radiation, and right SLF (*p* < 0.05, TFCE corrected; Figure 1; Table 2).

The RD of the middle cerebellar peduncle, genu of CC, body of CC, splenium of CC, right cerebral peduncle, right posterior limb of internal capsule, bilateral retrolenticular part of the internal capsule, bilateral superior corona radiata, bilateral posterior corona radiata, right posterior thalamic radiation, right sagittal stratum, right external capsule, bilateral cingulum (cingulate gyrus), right cingulum (hippocampus), right fornix/stria terminalis, and bilateral SLF was also significantly reduced in patients with KOA (*p* < 0.05, TFCE corrected; Figure 1; Table 2).

The overlapping white matter tracts of the FA, MD, AD, and RD were the body of CC and splenium of CC (Figure 1; Table 2). Using education level, SAS and SDS scores as covariates for further analysis did not change these results above with respect to only age, gender, and BMI as covariates.

### Correlations Between White Matter Tracts and Clinical Characteristics

**Figure 2:**
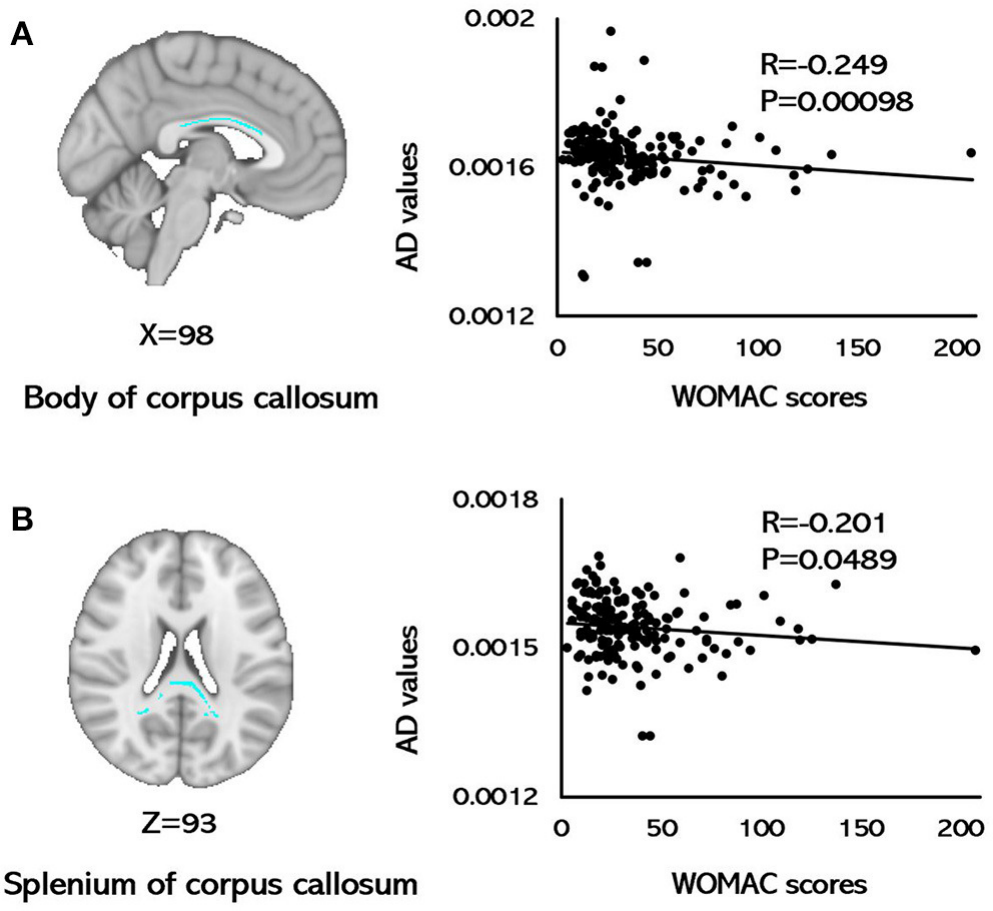
Correlations between AD values and WOMAC scores. **(A)** A negative correlation was observed between AD values for the body of the corpus callosum and WOMAC scores. **(B)** A negative correlation was observed between the AD values for the splenium of the corpus callosum and WOMAC scores (*p* < 0.05, FDR corrected). AD, axial diffusivity; FDR, false discovery rates; WOMAC, Western Ontario and McMaster Universities Osteoarthritis Index.

For the KOA group, AD values of the body of CC (*r* = −0.249, *p* = 0.0098; FDR corrected) and the splenium of CC (*r* = −0.201, *p* = 0.0489; FDR corrected) were correlated with WOMAC scores (Figure 2). None of the FA, MD, and RD metrics in any of the brain tracts was related with WOMAC, VAS scores, disease duration, SAS, or SDS (*p* > 0.05, FDR corrected).

## Discussion

This study featured a large sample size and used TBSS analysis to investigate alterations in the white matter of patients with KOA. Several regions in patients with KOA showed increased FA, and decreased MD, AD, and RD values when compared with HCs, including the CC, corona radiata, longitudinal fasciculus, cingulum, and thalamic radiation (Figure 1; Table 2). In patients with KOA, the AD values of the body of CC and splenium of CC were both correlated with WOMAC scores (Figure 2). These results reflected global white matter alterations in the KOA patients. To our knowledge, this is the first DTI study to detect alterations in the white matter of neural pathways in patients with KOA.

Functional changes in the regions of the brain responsible for perception, affection, and cognition have been detected in OA patients (12–14). These alterations in functional plasticity are often accompanied by gray matter remodeling and reorganization of the neurons, axons, and circuits (30, 31), further inducing the development and persistence of chronic pain (32). Furthermore, decreased gray matter volume (GMV) has been detected in the anterior cingulate cortex (ACC), orbital frontal cortex (OFC), lateral prefrontal cortex (PFC), precentral cortex, postcentral cortex, caudate nucleus, hippocampus, insula, thalamus, and amygdala of patients with OA (15, 16, 33). In this study, we found alterations in the white matter tracts of the CC, cingulum (cingulate gyrus), corona radiata, and superior longitudinal fasciculus in patients with KOA. These are all important components of the somatosensory and pain-related pathways and participate in the central integration and modulation of various peripheral perceptions, cognition, and emotion of pain (34).

The corpus callosum is the largest fiber tract and acts as a bridge for communicating perceptual, cognitive, volitional, and motor information between the two hemispheres (35, 36), and features prefrontal axons crossing the midline in the genu of the CC, somatosensory and motor axons crossing in the body of CC, and occipital and temporal axons crossing the midline in the splenium of CC (37). In the present study, we found that abnormal microstructure of the white matter spanned the length of the corpus callosum, thus suggesting alterations in the integration of cognitive, sensory, and motor information in KOA patients. Previous studies detected abnormal gray matter function and volume in the prefrontal, sensory, and cognitive regions in OA patients (12–16). Alterations in the CC connecting the sensory gyri might reflect an abnormal amount of nociceptive information entering the central nervous system from the peripheral nervous system. These alterations in motor integration may result from the evasive action evoked by KOA patients to lessen or avoid knee pain. The results of our study are in line with several other whole-brain TBSS studies which also found the abnormalities of the CC in patients with chronic pain diseases (25, 36, 38, 39). Furthermore, the AD values for the body of CC and the splenium of CC were negatively correlated with WOMAC scores in patients with KOA. Peripheral pathological pain is associated with persistent traumatic stimuli to the central nervous system and may be the microstructural basis for central sensitization, thus leading to central neuroinflammatory processes and edema (40, 41). Therefore, this correlation suggested that the integrity and neurofilament phosphorylation of axons in the CC may mediate individual variations in the clinical knee pain of patients with KOA. Abnormalities in the CC may be the specific indicator of maladaptive plastic modifications in KOA patients and CC-mediated interhemispheric connections might contribute to clinical sensory pain (42).

In the present study, we also detected an abnormal white matter microstructure in the corona radiata of patients with KOA. The corona radiata starts from the inner capsule and connects to the inferior frontal-orbital cortex and ACC, which is responsible for emotional expression and cognitive processing transmission between the brain hemispheres (43). Significant abnormalities in the corona radiata have been found in other chronic pain diseases, such as trigeminal neuralgia (39), chronic migraine (44), and chronic complex regional pain syndrome (CRPS) (45). These findings might suggest that there are abnormalities of emotional regulation in patients with chronic pain.

Increased FA values and decreased MD, AD, and RD values of the SLF were also found in patients with KOA in this study. Several previous studies have reported alterations in gray matter and abnormal functional brain activity in the insula, bilateral precentral gyrus, and frontal cortex in patients with chronic pain diseases, including chronic back pain (24), osteoarthritis (13, 14), and fibromyalgia syndrome (FMS) (25). Pain perception is mostly projected to the primary and secondary somatosensory areas, including the postcentral gyrus, paracentral lobule, precentral gyrus, and insula through the SLF (46). Furthermore, alterations in the microstructure of the white matter in the corticospinal tract were found in patients with KOA. The motor cortex may reduce the intensity of pain perception through the corticospinal tract; these represent the output pathway from cortical motor efferent to the descending pain modulatory system (47).

The cingulum is an important white matter pathway located within the limbic system (48). The midline and intralaminar thalamic nuclei (MITN) receive differing amounts of the spinothalamic tract, the pronociceptive sub-nucleus reticularis dorsalis, the parabrachial nucleus inputs, and project to the cingulate gyrus through the cingulum (49–51). In this study, microstructural alterations were mostly involved in the cingulum (cingulate gyrus) and cingulum (hippocampus). Persistent perceptive signals of pain lead to increased connections between the cingulum-hippocampal tract and default network, thus leading to the impairments in the avoidance behaviors provoked by OA (48).

Findings from TBSS studies of patients with chronic pain diseases are controversial (38, 52–55). In patients with pediatric migraine, carpal tunnel syndrome, and neuromyelitis optica spectrum disorders with neuropathic pain, the FA values were increased (53–55). Patients with idiopathic trigeminal neuralgia were found to have a lower FA, along with an increased MD, AD, and RD, in the white matter of connecting areas (52), while patients with migraine without aura showed lower MD and AD values in multiple white matter tracts of the brain (38). In the present study, we observed increased FA values but reduced MD, AD, and RD values in several white matter tracts in patients with KOA. There are several factors that may responsible for such discordances, including different kinds of diseases and subjects, sample sizes, research methods, scanning parameters, and statistical approach. Furthermore, the intensity and persistency of pain has been proven to be related with morphological and functional brain regions in patients with OA (12, 14). In this study, the mean VAS score of KOA patients was 4.31, which may have a milder effect on white matter than patients with a high intensity of pain. Also, we should consider that changes in neural expression of the white matter of patients with KOA might be related to a longer disease duration and concomitant neuroplasticity (40, 56). In this study, the mean disease duration of KOA was about 46 months. It is possible that central nervous system plasticity may have occurred after nerve impairment (56, 57). These changes in structural plasticity help pain-related learning and memory and may further contribute to the development of chronic pain or minimize the effects of pain on the body (57). In summary, the reasons for the controversial values of FA, MD, AD, and RD in white matter tracts reported in this study may be related to abnormal axonal integrity (axonal loss or the loss of bundle coherence) (58, 59), neural regeneration, and plasticity (56, 57).

There are several limitations in this study that need to be considered. First, this was a preliminary study relating to abnormalities of the white matter in patients with KOA compared with healthy controls. Second, correlations between the injury condition of the local knee joints and white matter alternations in KOA patients has not been identified. Third, the causation between alterations in the white matter tracts and KOA has yet to be elucidated. Longitudinal observation studies on the relationships between abnormalities in the white matter tracts and KOA need to be identified in further study.

## Conclusion

Patients with KOA showed extensive alterations in the white matter of the CC, corona radiata, longitudinal fasciculus, cingulum, and thalamic radiation. Furthermore, the AD values of the body and the splenium of CC were correlated with WOMAC scores in patients with KOA. Longitudinal observation studies on the causation between abnormalities in the white matter tracts and KOA are needed in the future.

## Data Availability Statement

The raw data supporting the conclusions of this article are available from the corresponding authors on reasonable request.

## Ethics Statement

The studies involving human participants were reviewed and approved by Institutional Review Board and Ethics Committees of the First Affiliated Hospital of Chengdu University of Traditional Chinese Medicine (No. 2016KL-017). The patients/participants provided their written informed consent to participate in this study.

## Author Contributions

FL, ZL, and FZ participated in the conception and design of the trial. SC, XD, JZ, CT, WH, and YC acquired the data. SC, XD, JZ, and XZ collated and analyzed the data. SC, XD, and JZ participated in drafting the manuscript. All authors contributed to the article and approved the submitted version.

## Funding

This work was supported by the National Natural Science Foundation of China (No. 81774400, 81973958, 81603708), Innovation Team and Talents Cultivation Program of National Administration of Traditional Chinese Medicine (No. ZYYCXTD-D-202003), China Postdoctoral Science Foundation (No. 2017M6100593, 2018T110954, PC2019012, 2021MD703796), and Medical Technology Project of Health Commission of Sichuan Province (No. 21PJ110).

## Conflict of Interest

The authors declare that the research was conducted in the absence of any commercial or financial relationships that could be construed as a potential conflict of interest.

## Publisher's Note

All claims expressed in this article are solely those of the authors and do not necessarily represent those of their affiliated organizations, or those of the publisher, the editors and the reviewers. Any product that may be evaluated in this article, or claim that may be made by its manufacturer, is not guaranteed or endorsed by the publisher.

## Abbreviations

3DT1: three-dimensional T1-weighted
ACC: anterior cingulate cortex
ACR: American College of Rheumatology
AD: axial diffusivity
BET: brain extraction tool
BMI: body mass index
CC: corpus callosum
CRPS: chronic complex regional pain syndrome
DTI: diffusion tensor imaging
FA: fractional anisotropy
FDR: false discovery rates method
FDT: FMRIB's diffusion toolbox
FMS: fibromyalgia syndrome
FOV: field of view
GMV: gray matter volume
HC: healthy control
IBS: irritable bowel syndrome
KOA: knee osteoarthritis
LPFC: lateral prefrontal cortex
MD: mean diffusivity
MITN: midline and intralaminar thalamic nuclei
MRI: magnetic resonance imaging
OA: osteoarthritis
OFC: orbital frontal cortex
PFC: prefrontal cortex
QOL: quality of life
RD: radial diffusivity
TR: repetition time
SAS: self-rating anxiety scale
SDS: self-rating depression scale
SLF: superior longitudinal fasciculus
TBSS: tract-based spatial statistics
TE: echo time
TFCE: threshold-free cluster enhancement
VAS: visual analogue scale
WOMAC: Western Ontario and McMaster Universities Osteoarthritis Index.
